# Optimal entropic properties of SARS-CoV-2 RNA sequences

**DOI:** 10.1098/rsos.231369

**Published:** 2024-01-31

**Authors:** Marco Formentin, Roberto Chignola, Marco Favretti

**Affiliations:** ^1^ Department of Mathematics Tullio Levi-Civita, University of Padova, via Trieste 63 35131 Padova, Italy; ^2^ Department of Biotechnology, University of Verona, Strada le Grazie 15-CV1, 37134 Verona, Italy

**Keywords:** Shannon entropy, mutual information, CT mutation bias

## Abstract

The reaction of the scientific community against the COVID-19 pandemic has generated a huge (approx. 10^6^ entries) dataset of genome sequences collected worldwide and spanning a relatively short time window. These unprecedented conditions together with the certain identification of the reference viral genome sequence allow for an original statistical study of mutations in the virus genome. In this paper, we compute the Shannon entropy of every sequence in the dataset as well as the relative entropy and the mutual information between the reference sequence and the mutated ones. These functions, originally developed in information theory, measure the information content of a sequence and allows us to study the random character of mutation mechanism in terms of its entropy and information gain or loss. We show that this approach allows us to set in new format known features of the SARS-CoV-2 mutation mechanism like the CT bias, but also to discover new optimal entropic properties of the mutation process in the sense that the virus mutation mechanism track closely theoretically computable lower bounds for the entropy decrease and the information transfer.

## 1. Introduction

With more than 270 million certified cases and nearly seven million deaths worldwide the COVID-19 pandemic caused by the severe acute respiratory syndrome coronavirus SARS-CoV-2 has had and is still having devastating consequences on our lifestyle, with impacts not only on human health but also on our social, political and economical systems [1]. Since the outbreak notified in Wuhan, China, in December 2019 the response of the scientific community has been immediate, and this global effort has quickly allowed us to decipher the biology of the virus thanks, in the first place, to the availability of modern and powerful sequencing techniques. These have provided a huge number of high quality sequences of the entire viral genome and of its variants, i.e. genomes that have arisen from the first viral genome through molecular evolution and fixed in the population by selection in infected hosts.

A wealth of information on viral genomes and on their phylogenetic relationships is indeed now available: importantly the sequence of the first virus isolated in Wuhan, i.e. the ancestor of the whole SARS-Cov-2 population, is known. It is therefore possible (and it is the aim of this work) to use mathematical tools from information theory to investigate quantitatively how the biological information stored in its genetic code has been transferred to the phylogenetically related variants and eventually find novel general principles.

Information theory was originally developed in the field of communication science to deal with problems such as the transmission of information from a source to a receiver over noisy channels and data compression, but it soon found applications in computational biology and bioinformatics, the main domains of applications being: alignment-free sequence analysis, study of sequence complexity, classification of motifs, prediction of transcription factor binding sites (see [2–6] for a recent and comprehensive review). Also coding theory has been used to gain insight into the DNA replication mechanism, see the pioneering papers [7,8] and the review papers [9,10]. Indeed, biological systems have a hierarchical organization that spans several orders of magnitude in both space and time, and biological information is transmitted back and forth across all levels through a multitude of molecular mechanisms, that are often error-prone and that take place in noisy environments. In particular, RNA viruses show high mutation rates because their RNA-dependent RNA polymerases—enzymes that are essential for nucleic acid duplication—unlike many DNA polymerases lack proofreading mechanisms and hence introduce errors in the sequence at a high rate during transcription [11]. Coronaviruses mitigate the effects of the low-fidelity polymerases by activating an exoribonuclease encoded in the nonstructural protein 14 (nsp14-ExoN) that proofreads RNA during replication through excision of mismatched incorporated nucleotides (see [12–14] and references cited therein). As a consequence, coronaviruses show a mutation rate which is intermediate between DNA viruses (10^−8^ to 10^−6^ substitutions per nucleotide site per cell infection; s n^−1^ c^−1^) and other RNA viruses (10^−6^ to 10^−4^ s n^−1^ c^−1^) [15,16]. Even a single mutation in the exoribonuclease can however result in the rapid accumulation of mutations in SARS-CoV-2 [17] showing that a complex balance between low-fidelity and proofreading mechanisms during RNA replication can drive SARS-CoV-2 evolution. In turn, high mutation rates together with short generation times and large population sizes cause accumulation of viral genetic diversity in the population and also within individual hosts.

Tools and concepts from information theory have been used to quantify viral genetic diversity within and between hosts even in the case of SARS-CoV-2 [18–20]. In general, however, the main focus of studies concerning SARS-CoV-2 variability has been the comparison of diversity at given sites along the nucleic acid sequence between viruses, above all within genome regions that are important for virus transmission and infectivity like the open reading frame coding for the spike protein (e.g. [21]). These efforts inevitably require the alignment of target sequences, a task that may become difficult for viral RNAs because of recombination and other rearrangement events that add diversity to single-nucleotide polymorphisms and that may hamper pairwise alignments [6,22]. Indeed, alignment-free sequence comparison has been recently remarked as a challenge in phylogenetic research, and applications of information theory have allowed the exploration of new fruitful methods and approaches [6].

Here we put forward a novel attempt to investigate viral diversity using concepts from information theory. The input data for the present study are the reference genome of SARS-CoV-2 (e.g. the Wuhan sequence NC045512.2) and its variants. These data are currently freely available in the National Center for Biotechnology Information (NCBI) public repository. We used this database because it provides high-quality and validated sequences along with curated metadata that can be exploited to filter and select the data on the basis of a variety of properties to obtain a homogeneous dataset (see Material and methods).

We compute the four-dimensional vector of the base frequencies of the reference genome sequence *x* and a variant *y* (called *q* and *p*, respectively). These input data are analysed using the main functions of information theory to compute the Shannon entropy *h*(*p*) of the variant, the relative entropy (also called the Kullback–Leibler divergence) *D*(*p*, *q*) between the reference genome and the mutated one, and the mutual information *I*(*x*, *y*) between the two genome sequences (see §4 for their definition and meaning). We are aware that reducing the complexity and the wealth of information contained in the RNA sequences to their base frequencies is a brutal simplification which prevents this study investigating any issues linked to functional domains of the genome or to virus fitness. Nevertheless, we show that this drastic simplification has allowed us to bring to light some new and unexpected optimal properties of the mutated SARS-CoV-2 sequences.

To start with, the entropy *h*(*p*) is a measure of the uncertainty or lack of information of *p*. Therefore, it is maximal when the four bases have the same frequency and it decreases when one of the bases in the sequence becomes prevalent. The frequencies of the four bases in the reference SARS-CoV-2 sequence are quite unbalanced in favour of A (*q*_A_/*q*_G_ = 1.52) and T (*q*_T_/*q*_C_ = 1.72). (Note: we use the tymine symbol instead of uracil for sequenced RNA genomes as in the NCBI database). It is well known that the rapid evolution of RNA viruses, including SARS-CoV-2, involves mutation processes that cause an asymmetry of C → T versus T → C transitions with a preponderance of C → T, the so-called CT bias [22,23]. Although the mechanistic bases of the CT bias are yet not fully understood, evidence points to the underlying role of mammalian antiviral mechanisms mediated by cytidine deaminase (deamination converts C to U) of the apolipoprotein B mRNA editing enzyme, catalytic polypeptide-like (APOBEC) family of proteins, that target the nucleic acids of viruses during their replication [22]. The role of these enzymes in inhibiting viral replication is well documented for retroviruses, such as human immunodeficiency virus, and certain DNA viruses like human papillomavirus [24]. In the case of other RNA viruses, SARS-CoV-2 included, APOBEC-mediated genome editing has been proposed to play a fundamental role for their long-term evolution [24]. Independently of its molecular explanation, we reasoned that the CT bias, if at work in the viral population, would unbalance still further the original viral genome with a consequent decrease of its entropy (i.e. informational content). In other words, the SARS-CoV-2 variants should show a decreased entropy with respect to their ancestor.

To investigate this phenomenon, we used the relative entropy *D*(*p*, *q*)—which is a measure of the dissimilarity between the frequencies *p* and *q*—as a proxy for the accumulation of mutations between the sequences *x* and *y*. Indeed in this work we show that the decrease in entropy has a computable theoretical lower bound and that there exist variants (mutated sequences) which are very close to the lower bound. For these sequences, we are able to give a theoretical formula for the base ratio, e.g. *p*_T_/*p*_C_ which shows a very good agreement with actual data (see §2.3).

A second line of investigation concerns the mutual information *I*(*x*, *y*) between the reference sequence *x* and a variant *y*. The mutual information is a nonlinear measure of the statistical coupling between *x* and *y* and it quantifies the amount of information about one variable obtained by observing the other. In other words, the higher the mutual information, the higher the fidelity of the RNA duplication mechanism in the sequence transcription. There is a theoretically computable trade-off curve (called a rate distortion curve) between the two competing objectives that allow us to minimize the mutual information for a given error threshold in the RNA sequence duplication. We computed the rate distortion curve for the variants evolved by the Wuhan sequence and again we find that there exists variants which reach this minimal information curve.

## 2. Results

In the first part of this section (§§2.1 to 2.5) we analyse the sequences in the dataset by plotting them in the entropy-relative entropy plane. We determine (§2.2) the theoretical minimum/maximum entropy curve and compute the fraction of sequences that are close to the minimum within a given error. In §2.3, we restrict our investigation to the entropy minimizing sequences of the same length as the reference sequence and in §2.4 we discuss a Markovian model which is capable of reproducing qualitatively the entropy decreasing character shown by these sequences. In the second part (§§2.6 and 2.7) for the restricted dataset of §2.3 we study the mutual information between the reference sequence and the mutated ones by computing for each sequence the matrix of transitions and trasversions. We plot the sequences in the mutual information-Hamming distance plane and in §2.7 we show that they are close to the minimum mutual information curve, called a rate distortion curve.

### 2.1. Relative entropy analysis

**Figure 1.**
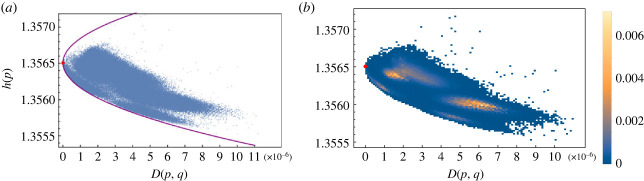
(*a*) Plot of entropy of variants of SARS-CoV-2 virus and minimum entropy curve. Red dot represents the reference sequence NC045512.2. (*b*) Density plot associated with figure in (*a*).

For every sequence in the dataset, we computed the frequency vector *p* = (*p*_A_, *p*_C_, *p*_G_, *p*_T_), its entropy *h*(*p*) and the ‘distance’ in relative entropy *D*(*p*, *q*) with respect to the frequency *q* of the reference sequence NC045512.2. Plotting the points (*h*(*p*), *D*(*p*,*q*)) a clear global pattern emerges: the entropy is decreasing with the distance *D*(*p*, *q*) from the reference genome, see figure 1*a*.

This shows that the accumulation of mutations (increasing *D*) drives away the frequency *p* from the uniform distribution where all the four bases are equally represented. This is a confirmation of the fact that single-nucleotide variations (SNV) are non-random and that a mutation bias exists. Indeed there is in the literature a consensus that in the SARS-CoV-2 genome mutations are strongly biased towards C → T transitions (see [23,25,26]). Another feature which is apparent from figure 1*a* is that the data points are organized in clusters. The identification of these clusters with known SARS-CoV-2 variants seems not to be unique (see the electronic supplementary material, figures S1-S5).

### 2.2. Existence of minimum entropy variants

Instead of focusing on a detailed biochemical explanation of the mutation bias, we have asked ourselves if the decrease in entropy shown by the variants has a computable theoretical lower bound. This amounts to solving the following constrained extremum problem: to find the probability *p* that has minimal entropy for a given value *D*(*p*, *q*) = *d* of the relative entropy distance. It turns out (see the electronic supplementary material for the explicit computation) that the extremal probability $\hat{p}$ is a function of the reference (Wuhan) frequency *q* of the form

2.1 $${\hat{p}}_{i}(\beta )=\frac{q_{i}^{\beta }}{Z(\beta )}=\frac{q_{i}^{\beta }}{\sum_{\;j\in \{A,C,G,T\}}q_{j}^{\beta }},$$

where the exponent *β* is determined by the constraint $D(\hat{p}(\beta ),q)=d$ and *Z*(*β*) is a normalization factor. The equation $D(\hat{p}(\beta ),q)=d$ has two solutions *β*^±^(*d*) corresponding to a maximal (*h*^+^(*d*)) and a minimal (*h*^−^(*d*)) value for the entropy. When *d* = 0 then *β* = 1 and we recover the reference distribution *q*. The upper and lower bound curves for the entropy are given by $h^{\pm }(d)=h(\hat{p}(\beta^{\pm }(d)))$. In figure 1, we have superposed the theoretical curve *h*^±^(*d*) to the points of the dataset. Note that the theoretical minimum/maximum entropy curves depend on the choice of the reference frequency *q* (see again the electronic supplementary material for the explicit computations).

**Figure 2.**
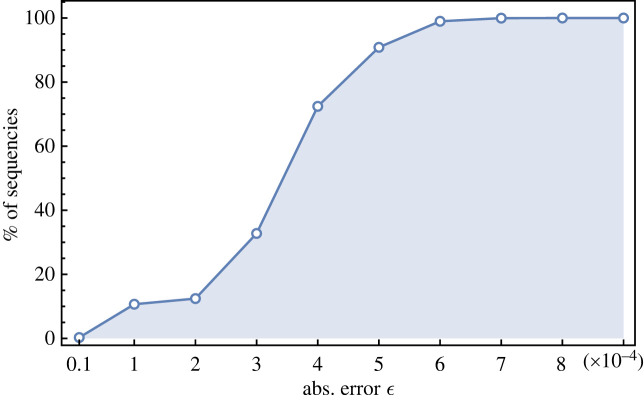
Plot of the percentage of sequences in the entire dataset that have entropy equal to the minimum within a fixed absolute error.

What we find with this study is the evidence that for SARS-CoV-2 coronavirus not only the entropy is decreasing but there exist variants (sequences) whose entropy is very close to the least possible value of entropy. Quantitatively, the following plot of figure 2 gives the number of sequences that have an entropy *h*(*p*) which is equal to the minimum value *h*(*d*) within a fixed absolute error ϵ. Note that the span in the value of entropy of the whole dataset is Δ*h* = (max *h* − min *h*) ∼ 10^−3^ (figure 1*a*). While it is understandable that the entropy decreases with *D* owing to the mutation bias briefly recalled before, it remains to be understood the origin of the ‘force’ that drives the variants to have the least possible entropy.

### 2.3. Quantitative analysis on a restricted dataset

To gain insight on the mutation mechanism and to provide quantitative estimates, we have restricted our analysis to a subset of the whole dataset comprised of the sequences which are (i) complete and without unknown characters, and (ii) have the same base length of the reference (Wuhan) sequence, i.e. 29903 bp. This restricted dataset (see Materials and methods) contains about 5600 sequences.

**Figure 3.**
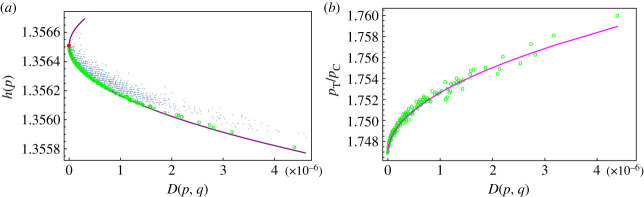
(*a*) Entropy of variants in restricted dataset, theoretical minimum/maximum entropy curves (violet); red dot represents the NC045512.2 (Wuhan) reference sequence, green dots are the running minima of minimal entropy variants. (*b*) Violet curve: *p*_T_/*p*_C_ ratio given by formula (2.2), green dots represent *p*_T_/*p*_C_ ratio for minimal entropy variants.

**Figure 4.**
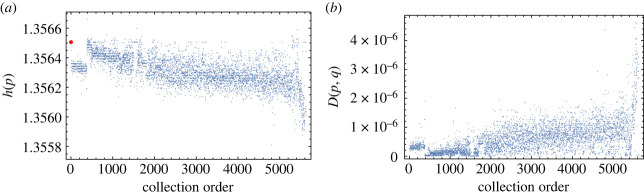
(*a*) Plot of entropy versus collection order for restricted dataset, and (*b*) plot of relative entropy versus collection order for restricted dataset.

As a preliminary step, to support the claim that the relative entropy *D*(*p*, *q*) is a good measure of the accumulation of mutations between the sequences *x* and *y* whose frequencies are *q* and *p,* we have compared the plot of *h*(*p*) versus relative entropy *D*(*p*, *q*) (figure 3*a*) with the plot of *h*(*p*) versus the collecting time *t* of the sequences (figure 4*a*). In both cases, the entropy is a decreasing function of *D* and *t* supporting the claim. In figure 4*b*, we show also that the relative entropy is an increasing function of the collecting time.

The most significant characteristic of the sequences in the restricted dataset is that they have entropy lower than their ancestor sequence *x* and they lie close to the minimum entropy curve. Note that the minimum entropy curve (the violet line in figure 3*a*) is the same in figure 1*a*.

An important application of formula (2.1) is that we can compute the base abundance ratio for variants that are close to the theoretical minimum entropy curve (lower branch of the violet curve in figure 3*a*) that we call variants of minimal entropy and that are highlighted as green dots in figure 3*a*. If we assume that the frequencies of these variants are reasonably well described by the formula (2.1) then the ratio in the abundance of two bases *i*, *j* at distance *d* is given by

2.2 $$b_{ij}(d)=\frac{\;p_{i}}{p_{j}}={(\frac{q_{i}}{q_{j}})}^{\beta^{+}(d)}.$$

Since *β*^+^(*d*) > 1, we know that if *q*_*i*_/*q*_*j*_ > 1 then the abundance ratio *increases* with *d*. Therefore the most abundant base will increase at the expense of all the others. We used the ratio *q*_T_/*q*_C_ = 1.72 between the most represented base (*q*_T_ = 0.320) and the least represented one (*q*_C_ = 0.186) in the reference sequence NC045512.2 and formula (2.2) to compute the violet curve in figure 3*b*. Even if this is an oversimplification of the real mutation process, the accord between the theoretical curve given by formula (2.2) and the value of the ratio *p*_T_/*p*_C_ for the variants of minimal entropy is surprisingly good (figure 3*b*).

The same investigation described above has been applied to the case of Mers virus of the coronavirus family. Even if the database comprises only 200 sequences, the results are comparable (see the electronic supplementary material, figures S8-S11).

### 2.4. Numerical model: Markov chain

The patterns illustrated in figure 3*a* can be obtained through simple discrete-time Markovian dynamics with the following rules: (i) the sequence at time zero is the reference sequence NC045512.2; then (ii) a base *i* ∈ {A, C, G, T} of the sequence is chosen at random; and (iii) the base *i* picked in step (ii) mutates into *j* ∈ {A, C, G, T} with probability *m*_*ij*_. At each subsequent time step, rules (ii) and (iii) are applied to the mutated sequence.

In order to estimate the transition matrix *M* = (*m*_*ij*_)_*i*,*j*∈{A,C,G,T}_, the ideal would be to have a large sample of one-step trajectories of the Markovian dynamics described above. The endpoints of these trajectories would be sequences where only one base has been changed by the dynamics. In our dataset, the best proxy of this set of sequences is the set of sequences at the smallest Hamming distance [27] from the reference sequence. Thus, we determined the transition matrix *M* = (*m*_*ij*_)_*i*,*j*∈{A,C,G,T}_ as follows: we computed the normalized Hamming distance from the reference sequence NC045512.2 for each sequence in the restricted dataset and considered the set *W* of the sequences having the smallest distance from the reference sequence.

Then for *i*, *j* ∈ {A, C, G, T} we define *n*_*ij*_(*x*, *y*) to be the number of bases that change from *i* in the reference sequence *x* into *j* in *y* ∈ *W* and $n_{i}(x)=\sum_{j}n_{ij}(x,y)$. Finally, we compute the transition matrix *M* as the empirical mean

$$m_{ij}=\frac{1}{\left|W\right|}\sum_{y\in W}\frac{n_{ij}(x,y)}{n_{i}(x)},$$

where |*W*| is the cardinality of *W*. The matrix *M*, up to an error of order 10^−6^, reads as follows:

$$M=\left(\begin{array}{cccc} 0.99999 & 0 & 1\times 10^{-5} & 0\\ 1\times 10^{-5} & 0.99993 & 0 & 6\times 10^{-5}\\ 0 & 0 & 0.99995 & 5\times 10^{-5}\\ 2\times 10^{-5} & 0 & 0 & 0.99998 \end{array}\right).$$

**Figure 5.**
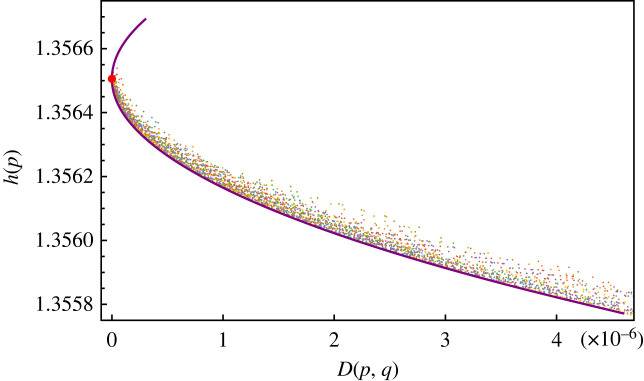
Entropy versus relative entropy plot for the sequences generated using the Markov model. Starting from the reference sequence NC045512.2 we ran the model for 10^6^ steps. We ran the model 100 times assigning to each run a different colour. The generated sequences closely follow the minimum entropy branch.

**Figure 6.**
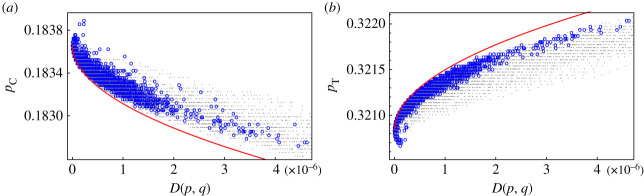
Plot of the frequency *p*_C_ (panel (*a*) on the left) and *p*_T_ (panel (*b*) on the right) versus relative entropy. Blue circles represent sequences of the restricted dataset superimposed to the frequencies of the sequences from the numerical simulations of the Markov model (grey dots). Red curves represent theoretical maximum/minimum frequency curves. The quantity *p*_C_ decreases while *p*_T_ increases with the relative entropy *D*(*p*, *q*).

Such a simple Markov model generates a entropy versus relative entropy pattern that resembles the one found for the SARS-CoV-2. In particular, the entropy of the sequences follows closely, as the relative entropy increases, the minimum entropy curve (figure 5). Numerical simulations suggest the key feature for the pattern in figure 5 to appear is that the transition matrix favours the mutation C → T and A → G enhancing the imbalance between the base frequencies *p*_C_ and *p*_T_, and *p*_A_ and *p*_G_ of the reference genome. Indeed for the reference sequence NC045512.2 we have *q*_T_/*q*_C_ = 1.72 and *q*_A_/*q*_G_ = 1.52. Such a behaviour is confirmed by the analysis of the restricted dataset: *p*_T_ increases with the relative entropy while *p*_C_ decreases, providing evidences of the bias C → T. See figure 6 for a comparison between the data and the model.

We remark that, within our Markov model, to substitute *W* with sequences at larger Hamming distance—which would be the results of several steps of the dynamics—would correspond to estimate a power of the transition matrix M, rather than M itself. In the electronic supplementary material, figures S12 and S13 we investigated how the entries of the transition matrix change when one considers sequences at a larger distance in place of *W*. Off-diagonal terms, in particular the entries CT and GT, increase with larger Hamming distance.

### 2.5. Maximum frequency variants

Looking at figure 6, we see that the frequencies *p*_C_ and *p*_T_ of the variants in the restricted dataset (blue circles) form a pattern with a neat front. Reasoning as we did in §2.2 we can compute a theoretical curve describing the front; this amounts to find the probability distribution that maximizes *p*_T_ (respectively, minimize *p*_C_) for a given value of relative entropy *D*(*p*, *q*) = *d* (red curves in figure 6). See the electronic supplementary material for explicit computations.

### 2.6. Mutual information analysis

A second line of investigation concerns the mutual information *I*(*x*, *y*) between the reference *x* sequence and a variant *y*. If we think of *x* and *y* as realizations of random variables *X* and *Y*, then the mutual information *I*(*X*, *Y*) = *h*(*X*) − *h*(*X*|*Y*) is defined as the difference between the entropy of *X* and the conditional entropy *h*(*X*|*Y*) of *X* given *Y*. So the mutual information is the reduction of uncertainty in *X* given by the knowledge of *Y*. It is a symmetric, nonlinear measure of the degree of statistical coupling between the two variables and it quantifies the amount of information obtained about one variable by observing the other, therefore *I* is zero when the two variables are statistically independent. In the mathematical theory of communication, *x* and *y* represent the input and output sequence of a message which is sent through a noisy communication channel. An efficient channel is the one that minimizes *I* assuring that the error between the input and output message does not exceed a given threshold.

Mutual information has been applied in genetic studies to estimate pairwise correlations between gene expressions [28,29] or gene network reconstruction [30,31] and genetic distance measure [32]. Within rate-distortion theory, it has been applied to provide a model of genome sequence evolution and to compute rate-distortion curves [33] or describe biological signalling [34,35]. This approach has also been applied in other domains (rational inattention theory in economy [36,37]) or in human perception studies [38].

The constrained extremum problem (minimize *I* for a given error threshold) introduced above can be used to investigate the duplication process of nucleic acids. In our application to the analysis of viral sequences, we do not have random variables *X*, *Y* but RNA sequences *x*, *y* which have the same length *n* and whose empirical frequencies *q*, *p* are known. The mutual information between *x* and *y* can be written as (see the electronic supplementary material):

2.3 $$I(x,y)=I(q,P)=\sum_{i,j}q_{i}P_{ij}\ln\left(\frac{P_{ij}}{\sum_{k}q_{k}P_{kj}}\right),$$

where *q*_*i*_ = *n*_*i*_(*x*)/*n*, *P*_*ij*_ = *n*_*ij*_(*x*, *y*)/*n*_*i*_(*x*) is the conditional probability and *n*_*ij*_(*x*, *y*) is the count of the transition–transversions between the reference sequence *x* and a variant *y*.

Note that *I*(*x*, *y*) is not a function of the sole frequencies *p*, *q* like the functions *h*(*p*) and *D*(*p*, *q*) used above because it also depends on the matrix of transitions and transversions *P*. Another difference with respect to the entropy-relative entropy (*h*, *D*) plane analysis is that now we measure the discrepancy between the virus sequences *x* and *y* using the Hamming distance *d*_H_(*x*, *y*) which is the number of corresponding sites where the two sequences differ by an SNV, see [27]. This requires that all the sequences have equal length *n*. The Hamming distance is a finer measure of the dissimilarity between two sequences because *d*_H_ is non-zero when two sequences differ by a simple permutation of the sites while the relative entropy is non zero only if the two sequences have different base frequencies.

In mathematical terms, given *q*, we want to find the conditional probability matrix *P* = *P*(*d*) which minimizes the mutual information *I*(*q*, *P*) subject to the constraint *d*_H_(*q*, *P*) = *d*. The minimal mutual information is a curve *I* = *I*(*d*) (see the electronic supplementary material, formula S13) called the rate function.

### 2.7. Existence of minimal mutual information variants

**Figure 7.**
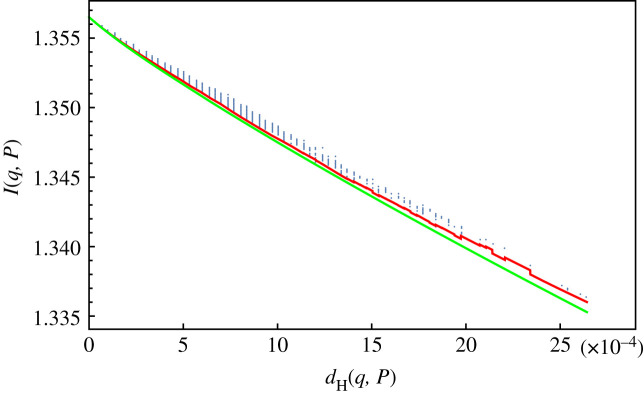
Blue points: mutual information *I*(*q*, *P*) of a variant in restricted dataset as a function of Hamming distance; green line: minimal information curve for *r* = 2; red line: minimal information curve with variable *r* = *r*(*d*).

For every variant *y*, we have computed the Hamming distance *d*_H_(*x*, *y*) with respect to the reference sequence *x*, the matrix *P*_*ij*_(*x*, *y*) described above and the mutual information *I*(*x*, *y*). We have plotted the *I*, *d* points in the plane and we have superimposed to the plot the curve *I*(*d*) which gives the minimal possible value of *I* for a given value of the Hamming distance, see figure 7. We find that there are variants *y* which have mutual information very close to the minimal possible value. If we model the RNA duplication problem as the problem of reliable transmission of a sequence over a noisy (i.e. error prone) channel, then the above result suggests that the RNA viruses replication process minimizes the degree of coupling between the input and output to achieve a given error threshold.

A possible explanation of the above result could be as follows: the error correction performed by the kinetic proofreading [39,40] mechanism in the duplication of nucleic acid is, from the point of view of the thermodynamics of information [41], a logically irreversible operation [42]. Therefore, in accordance with Landauer principle [43] it must be accompanied by an entropy increase in the system or in the environment. In particular, the work performed in a measurement (state recognition) and erasure (error correction) process is greater or equal to the mutual information between the template and the copy (see again [41,44–46]). So it seem plausible to link the minimization of mutual information between the source and output with the minimization of the thermodynamic cost of correcting the transcription errors.

One can observe that the theoretical minimal mutual information curve has a poorer fit with the experimental points in the lower part of the plot in figure 7. In the electronic supplementary material, we show how we can obtain a better fit adding a constraint on the ratio *r* = *Tv*/*Ti* between transversions and transitions. The conclusion is that the sole knowledge of the ratio *r* is enough to determine the matrix *P* of the base substitutions for the variants that display the optimal behaviour.

## 3. Discussion

In this work, we carried out a statistical study on the sequences of SARS-CoV-2 (see also [47,48] for an early attempt in this direction). The unprecedented conditions of a very large (approx. 10^6^ records) dataset spanning a short time window (3 years) and a clearly identified ancestor (the Wuhan NC045512.2. sequence) allows for a novel study of the genome mutations. Instead of focusing on the most relevant loci of the genome to study particular virus characteristics we have adopted an all-genome analysis using: (i) the simplest level of description, the sequence base frequency *p*, and (ii) two statistical indicators: the entropy *h*(*p*) of the sequence and the relative entropy *D*(*p*, *q*) between the frequency *p* of a generic sequence in the dataset and the frequency *q* of the reference sequence NC045512.2. Note that *D*(*p*, *q*) is zero if *p* = *q* and it increases with the accumulation of the number of mutations in the sequence. The entropy *h*(*p*) is a measure of the uncertainty associated with *p* and it is maximal when the four base frequencies are equal. A mutation in the genome induces a tiny variation in the base frequency *p* with respect to *q* which can result in *h*(*p*) > *h*(*q*) if *p* is more ‘uniform’ than *q* and in *h*(*p*) < *h*(*q*) if the mutation increase the unbalance between the base frequencies. We find that for the overwhelming majority of the sequences the mutations *decrease* the entropy. A plot of the base frequencies shows that the unbalance *p*_T_ > *p*_C_ (and on a smaller scale also for *p*_A_ > *p*_G_) is increased with respect to *q*_T_ > *q*_C_, a phenomenon already reported in literature, the so-called CT bias. A new feature emerging with our approach is that for a minority of the sequences in the dataset the decrease in entropy reaches a theoretically computable lower bound, i.e. for a given level of mutation (measured by *D*(*p*, *q*)) the mutation mechanism is capable of reaching the maximum possible value of the unbalance. A detailed biochemical explication of this result seems to be beyond the reach of the approach adopted in this study.

Apart from the one described above, the mutation mechanism of SARS-CoV-2 seems to display a most efficient behaviour in another respect. This is demonstrated in our study using the mutual information *I*(*x*, *y*) between a mutated sequence *y* and the ancestral one *x*. It is a measure of the reduction of the uncertainty about *x* allowed by the knowledge of *y,* therefore it is zero if *x* and *y* are totally uncorrelated and it is maximal when the sequence *y* is a copy free of errors of *x*. Therefore, the replication mechanism is maximally efficient when *I* is minimal for a fixed error threshold. Again, what we find with this study is that there are mutated sequences for which the mutual information is close to the theoretical lower bound, the so called rate-distortion curve. Even if a complete explanation of this optimal (in the sense of most efficient) behaviour of the virus mutation in terms of first principles seems still lacking we think that this novel approach could be a valid complement to the more functionally oriented analysis of RNA mutations.

A key point of our study is that all the statistical functions used depend on the precise individuation of an ancestral sequence *x* from which the base frequency *q* can be computed. This fact prevents the study of subsets of the dataset (for example the set of sequences classified as the specific variant Omicron) if the sequence originating the specific variant is not known.

The statistical functions and the overall approach discussed in this paper might nonetheless be exploited to investigate not only the evolution of whole genomes, as we show here, but also that of individual genes or more generally of interesting genetic regions. For example, the frequencies of dinucleotides in a given RNA region might be taken into account to quantitatively study the landscape of CpG sites that are involved in virus evolution, replication and host immune response (since CpG are pathogen-associated molecular patterns recognized by the innate immune system, [49]). This landscape has been recently shown to vary heterogeneously along the genome in response to virus adaptation to evolutionary pressure [50,51]. A straightforward extension of our equations might also allow us to investigate the evolutionary landscape of viral molecular phenotypes, i.e. of amino acid sequences on which the selective forces that drive evolution operate. Proteins are sequences written in a 20—instead of four—letter code, after all. We therefore hope that the results presented here may complement more functionally oriented kind of analysis of the mutation mechanism and stimulate the research about the fundamental ‘laws’ that control the efficiency of the mutation mechanism and ultimately molecular evolution.

## 4. Material and methods

Genome sequences were retrieved from the NCBI database (https://www.ncbi.nlm.nih.gov). The sequences were filtered according to the following criteria:

(i) *SARS-Cov-2 dataset*: we selected the complete genome sequences from a human host, with none unknown characters. in the NCBI database, there are about 950 000 sequences with these characteristics;
(ii) *restricted SARS-Cov-2 dataset*: we selected the complete genome sequences from a human host, with none unknown characters, with 29 903 bases, that is of the same length as the reference sequence NC045512.2. We obtained, from the NCBI database, about 5600 sequences with such features. From this set we deleted 15 sequences having a normalized Hamming distance larger than 0.5 from the reference sequence; and
(iii) *Mers dataset:* we selected the about 200 complete genome sequences from a human host, with none unknown characters. For our analysis, we used NC019843 as the reference sequence.

Sequences have been processed through a C++ code (provided with the datasets) that reads a dataset of nucleic acid sequences in FASTA format and returns the number of bases in each sequence. The output file contains a table organized as follows: first column, number of bases in a given sequence; second, third, fourth and fifth columns, number of bases of type A, C, G and T, respectively, in the same sequence. Each row reports the data calculated for successive sequences following the same order of the raw datasets. Processed data are available at the following link: https://doi.org/10.5061/dryad.9s4mw6mp2 [52].

### 4.1. A synopsis of information theory functions

See [53, ch. 1] for a gentle introduction to entropy and relative entropy. The Shannon entropy of a discrete distribution *p* is $h(p)=-\sum_{i}p_{i}\ln p_{i}$. It is a measure of the uncertainty or lack of information on the system statistically described by *p*. The entropy is maximal when *p* is the uniform distribution and minimal when *p* is concentrated at a single state. Let *q* and *p* be two probability distributions.

The relative entropy, also called Kullback–Leibler divergence, is a statistical distance of the form:

4.1 $$D(p,q)=\sum_{i}p_{i}\ln\frac{\;p_{i}}{q_{i}}=-h(p)+\sum_{i}p_{i}\ln\frac{1}{q_{i}}.$$

The relative entropy is a measure of the gain in information when one statistically describes the system with *p* assuming that the ‘true’ description is given by *q*. *D*(*p*, *q*) ≥ 0 if *p* ≠ *q* and *D*(*q*, *q*) = 0. Even if *D* does not satisfies the requirements of a distance function, it is a measure of the discrepancy between *p* and *q*.

Let us denote with *X* ∼ *p*_*X*_ and *Y* ∼ *p*_*Y*_ two random variables. It is customarily to write *h*(*X*) and *h*(*Y*) for *h*(*q*) and *h*(*p*). Let us denote with *p*_*XY*_ the joint distribution of *X* and *Y*. The conditional entropy of *Y* known *X* is [53]:

$$h(Y|X)=\sum p_{XY}\ln\frac{\;p_{XY}}{p_{X}},$$

and it represents the uncertainty about *Y* when *X* is known. Accordingly:

4.2 I(X,Y))=h(X)−h(X|Y),

called *mutual information* of *X*, *Y* represents the reduction of uncertainty on *X* owing to the knowledge of *Y*. Note that when *Y* = *X*, *h*(*X*|*X*) = 0 and *I*(*X*, *X*) = *h*(*X*) therefore the entropy *h*(*X*) is also called self-information. *I*(*X*, *Y*) is a symmetric and non-negative function of *X*, *Y* because it can be rewritten as a relative entropy:

$$I(X,Y)=D(p_{XY},p_{X}p_{Y})=\sum p_{XY}\ln\frac{\;p_{XY}}{p_{X}p_{Y}}.$$

From the above formula, it is apparent that the mutual information is zero when the two variables are independent *p*_*XY*_ = *p*_*X*_*p*_*Y*_. Indeed the mutual information is a nonlinear measure of the statistical coupling between the variables which constitutes a generalization of the covariance *c*(*X*, *Y*). In communication theory, the mutual information is called the rate function.

## Data Availability

We uploaded on Dryad (https://doi.org/10.5061/dryad.9s4mw6mp2) three datasets: (i) SARS-Cov-2 dataset. This dataset contains number of bases for the complete genome sequences from a human host, with none unknown characters. In the NCBI database, there are about 950 000 sequences with these characteristics; (ii) restricted SARS-Cov-2 dataset. This dataset contains number of bases for the complete sequences from a human host, with no unknown characters, with 29 903 bases, that is of the same length as the reference sequence NC045512.2. We obtained, from the NCBI database, about 5800 sequences with such features; and (iii) Mers dataset. This dataset contains number of bases for the complete sequences of about 250 complete genome sequences from a human host, with no unknown characters. The above datasets were obtained from raw genome sequences retrieved from the NCBI database (https://www.ncbi.nlm.nih.gov), processed through a C++ code (provided with the datasets) that reads a dataset of nucleic acid sequences in FASTA format and returns the number of bases in each sequence. The output file seqcount.txt contains a table organized as follows: first column, number of bases in a given sequence; second, third, fourth and fifth columns, number of bases of type A, C, G and T, respectively, in the same sequence. Each row reports the data calculated for successive sequences following the same order of the raw datasets [52]. Data is also provided in the electronic supplementary material [54].
